# Noise filtering and nonparametric analysis of microarray data underscores discriminating markers of oral, prostate, lung, ovarian and breast cancer

**DOI:** 10.1186/1471-2105-5-185

**Published:** 2004-11-29

**Authors:** Virginie M Aris, Michael J Cody, Jeff Cheng, James J Dermody, Patricia Soteropoulos, Michael Recce, Peter P Tolias

**Affiliations:** 1 Center for Applied Genomics, Public Health Research Institute, Newark, NJ 07103, USA; 2 Center for Computational Biology, New Jersey Institute of Technology, Newark, NJ 07103, USA; 3 Dept of Microbiology and Molecular Genetics, UMDNJ-New Jersey Medical School, Newark, NJ 07103, USA; 4 Current address: Ortho-Clinical Diagnostics a Johnson & Johnson Company, Raritan, NJ 08869, USA

## Abstract

**Background:**

A major goal of cancer research is to identify discrete biomarkers that specifically characterize a given malignancy. These markers are useful in diagnosis, may identify potential targets for drug development, and can aid in evaluating treatment efficacy and predicting patient outcome. Microarray technology has enabled marker discovery from human cells by permitting measurement of steady-state mRNA levels derived from thousands of genes. However many challenging and unresolved issues regarding the acquisition and analysis of microarray data remain, such as accounting for both experimental and biological noise, transcripts whose expression profiles are not normally distributed, guidelines for statistical assessment of false positive/negative rates and comparing data derived from different research groups. This study addresses these issues using Affymetrix HG-U95A and HG-U133 GeneChip data derived from different research groups.

**Results:**

We present here a simple non parametric approach coupled with noise filtering to identify sets of genes differentially expressed between the normal and cancer states in oral, breast, lung, prostate and ovarian tumors. An important feature of this study is the ability to integrate data from different laboratories, improving the analytical power of the individual results. One of the most interesting findings is the down regulation of genes involved in tissue differentiation.

**Conclusions:**

This study presents the development and application of a noise model that suppresses noise, limits false positives in the results, and allows integration of results from individual studies derived from different research groups.

## Background

DNA microarrays have become a useful tool in biomedical research as they can be used to determine the relative expression of thousands of genes in a given sample. Such expression profiles could predict genetic predisposition to disease, serve as a set of diagnostic markers, define better drug treatments options for existing diseases (pharmacogenomics), or mark the precise nature of disease progression. A major limitation of this technology is the lack of uniform data mining strategies. This study integrates complementary approaches to more effectively analyze Affymetrix GeneChip microarray data derived from several different types of solid tumors. If the noise is consistent and reproducible it can be filtered from the data and some false positives can be eliminated. There are two principal sources of noise in microarray experiments: biological noise and technical noise. Biological noise consists of variation among patients and tumor locations, variation in the cellular composition of tumors, heterogeneity of the genetic material within tumor due to genomic instability. Technical noise consists of differences in sample preparation and experiment variables which include nonspecific cross hybridization, differences in the efficiency of labeling reactions and production differences between microarrays. Biological noise cannot be corrected but it can be accounted for with statistics using replicates of the treatments or conditions. However, the noise derived from experimental techniques is reproducible and its boundaries can be modeled. It has been observed that in differential gene expression comparisons of any given gene, there is a greater variance in the fold-change calculation at lower signal intensities [1,2], and when comparing replicate samples, lower expression values tend to have greater variance in signal intensity. This suggests that larger errors can occur when lower signals are used to compute fold-changes in differential comparisons. Fold change, computed in this way, can lead to extraneous inclusions in lists of significantly up-regulated or down regulated genes. For example, a fold change of two calculated from intensities of 25 and 50 may not be as trustworthy as a fold-change of two determined between intensity values of 2,000 and 4,000. Thus, the purpose of error boundary modeling is to reduce the influence of less trustworthy fold-change calculations in the analysis of differential microarray data. The efficacy of coupling a noise boundary model to an analysis method has been previously shown for two color cDNA arrays [3-6].

The principal concerns when using microarray data derived from different labs to identify cancer markers is that chip-to-chip normalization cannot eliminate differences in cRNA synthesis and labeling, hybridization protocols, scanner settings and image processing software. Variable RNA quality can influence the amount of individual cRNAs generated. The laser power on scanners can differ causing saturation of high intensity probe sets and may have a more variable estimation of the very low expressed transcripts.

Two studies [7,8] have successfully classified different types of cancer by their molecular profile on microarrays using hierarchical clustering and support vector machine (SVM) techniques. Both studies found that their markers comported a high number of genes whose expression differed among the normal tissues of origin. The approach taken in this paper is that the cancer samples are compared first to their normal tissue and then the most discriminating genes for each cancer vs. normal comparison are compared between cancers. This circumvents normalization problems due to lab-specific parameters (scanner settings, labeling, hybridization variables) and tissue specific artifacts, as each cancer biopsy is compared to its corresponding normal tissue processed by the same research group, in the same environment. These environmental parameters and artifacts are assumed to be similar for the normal and cancer biopsies and should cancel out. This allowed the selection of the genes that best discriminated between the normal and tumor samples. These classifiers were then evaluated to see if they were specific to the different types of cancers. Since gene expression measurements of individual Affymetrix GeneChips probe sets frequently do not follow a normal distribution, a non-parametric analysis was used.

The commonly used t-test tests to see if two populations have a different mean but does not test the overlap of the populations. Selecting markers with minimal overlap in their expression between the normal and tumor states would improve their predictive value. We developed a method to find such markers using an un-weighted voting scheme. This non-parametric method for marker selection was chosen so that no assumptions on the shape of the data distribution were required. The computed noise boundary makes the selection criteria more stringent, eliminating many false positives signals and highlighting genes that are differentially expressed most consistently in comparisons between a cancer and its corresponding normal tissue. This integrative approach can yield a signature of distinct transcripts distinguishing a variety of solid tumors.

The objectives of this work were three fold: 1). develop a noise boundary for GeneChip data, 2). develop an algorithm for selecting markers with minimal overlap in their expression between the normal and tumor states, 3). integrate the analysis of previously published data from different sources.

## Results and discussion

### Noise boundary model

The noise boundary model was created to evaluate the reliability of calculated fold changes. Data from normal tissue biopsies obtained from public data sets were used to infer the noise boundary to be used when comparing the normal tissue to their corresponding cancer biopsies. The cancer biopsies were not used in designing the noise model as they are likely to be more variable than normal tissue. Three different signal extraction methods, MAS5 [9,10], dChip [11,12] and RMA [13] were compared and MAS5 was chosen as it showed stable results and is widely used. A complete analysis of the three methods is included in the supplemental data [see Additional file 2].

**Figure 1:**
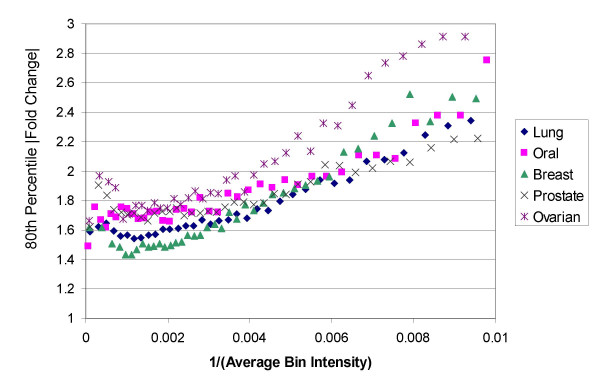
This figure represents the 80^th ^percentile for each of the five normal tissues plotted against the inverse of the average bin intensity. Bins with an average intensity below the cutoff of 100 (above 0.01 in the figure) were not displayed as they are below the minimum intensity cutoff.

A fold-change threshold boundary was drawn for each comparison between normal tissues for each of the cancers studied to model the noise inherent to the method. The data was first sorted according to the average intensity of the values of the probe-sets for two replicate chips. If there is no noise in the technique or the biology, one should expect to have all the fold changes be 1. However when plotting the fold changes against the average intensity for the probe-sets we observed that the data formed a volcano plot with considerable scattering for low intensity and a progressive tapering of the fold changes at high intensities. As there is a lot of noise in estimating the low end expression a cut-off is needed to eliminate part of that noise. Then, as the samples were biological replicates, we assumed that most of the genes were not differentially expressed; a certain percent of the genes should not change and a percentile cut off was set up to eliminate spurious variations. The data was then binned into fixed width bins including 200 expression values. For modeling purposes, the percentile was plotted against the inverse of the average bin intensity to reveal a linear relationship that can be characterized with a slope and intercept. A sensitivity analysis to optimize the noise boundary percentile and low intensity cut off parameters was performed and is presented in the supplemental data [see Additional file 2]. The low intensity cutoff was set to 100 and bins with a mean expression value lower than 100 were excluded. The 80^th ^percentile of the fold-change, chosen as the noise boundary, was calculated for each bin. Figure 1 shows the 80^th ^percentile error boundaries for the five different tissues as a function of the inverse of the average bin intensity. To decrease the effect of saturation on the regression, the gene expression values in the top 8% were eliminated (this correspond to the 5 highest bins intensities in figure 1). The noise boundary was found to be tissue dependant and the slope and intercept were calculated for each tissue (Table 1). For a fold change to be considered reliable, it has to be greater than the noise boundary threshold for the same average intensity:


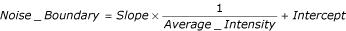


### Nonparametric microarray data analysis: Er Algorithm

In microarray experiments, the number of replicates is often small and the distributions of gene expression are not normal for all genes. For the same difference in mean, depending on the distribution of the data, the overlap of two distributions can be dramatically different. Ideal markers would be genes with no overlap in their distribution; the consistency of change is therefore more significant than the amplitude. To address the problem of low numbers of replicates and multiple testing on 12,000 genes, the noise boundary model was incorporated with non-parametric data mining. The noise boundary eliminates noise that is proportional to the probe intensity measured. The combination of the non-parametric voting scheme with the noise model will be referred to as the directional change assessment algorithm. For each transcript, the ratio of expression intensities (fold change) of each cancer biopsy to each normal biopsy was determined. Those ratios were recorded and evaluated against the noise boundary model. If a ratio was above the ratio given by the noise boundary, the direction of the fold-change as increased (+) or decreased (-) expression was recorded. If the ratio was below the value given by the noise boundary for the average of the intensities, then the fold-change was considered insignificant and assigned a no change (0) direction.

For each probe-set

For each sample c_i _in the cancer class

For each sample n_j _in the normal class

If c_i>_n_j _then r = c_i_/n_j _else r = n_j_/c_i_

If r>noise_boundary ((c_i_+n_j_)/2) And If c_i>_n_j_

Pos_score = Pos_score + 1

If r>noise_boundary ((c_i_+n_j_)/2) And If c_i _≤ n_j_

Neg_score = Neg_score + 1

Else NoChange_score = NoChange_score + 1

We designed an index, called event ratio to summarize the overlap in distribution between the cancer and normal samples. This Er index is described by:


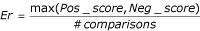


Where the #comparisons is equal to the number of cancer samples multiplied by the number of normal samples. This method counts direction and evaluate the overlap of the distributions normalized to the number of comparisons. The Er index ranges from 0 to 1. As the direction of change for a gene becomes more consistent, Er approaches 1. Conversely, if the Er score is close to 0.5, then the gene is inconsistent with regard to its directionality and thus cannot be considered a reliable marker for disease classification. As the score approaches 0, the transcript direction and fold change cannot be reliably estimated as it is within the noise level of the technique. The software is available at .

**Figure 2:**
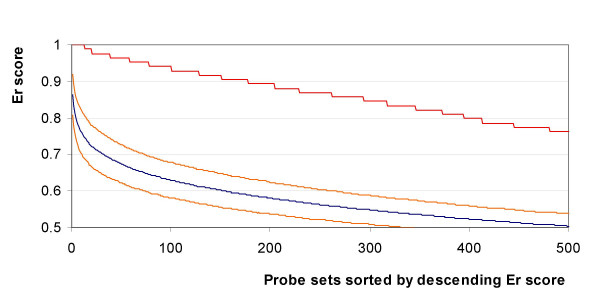
Comparison of the Er score of the 500 top ranked probe sets for breast cancer versus normal breast biopsies. Er score for the real breast cancer vs. normal biopsies (red line), Average Er score of the 500 top ranked probe sets of the 100 shuffling sets (blue line), one standard deviation away form the average shuffled sets (orange line).

**Figure 3:**
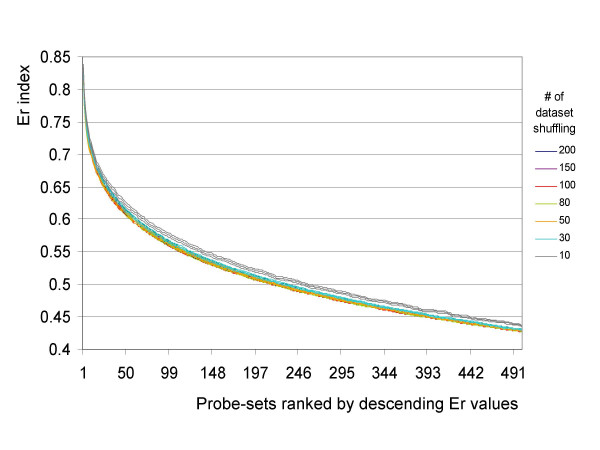
Average Er scores for the breast shuffled sets depending on the number of shuffling. The average Er score and a standard deviation above and below are represented for 10, 30, 50, 80, 100, 150 and 200 shuffling of the dataset. We can see that the average Er score converges rapidly after 50 shuffling of the data set.

To test the validity of this approach, the samples were shuffled 100 times between the categories (cancer and normal) and the Er computation was repeated. Each time the data was shuffled, the probe sets were sorted by descending Er score and the probe set information was discarded and replaced by its rank. The average and standard deviation of the ranks was then computed and compared to the results obtained for the cancer versus normal biopsies. For all the comparisons performed, higher Er scores were obtained in the case of cancer versus normal classifications than with randomly shuffled sets. An illustration of the results obtained with the breast cancer versus normal biopsies can be seen in figure 2. The average Er score per rank converged rapidly, and was consistent after shuffling the dataset 50 times (figure 3).

### Comparison of the Noise Boundary-Er Algorithm to standard analysis techniques

To compare the Er algorithm including the noise model to other commonly used analysis methods, the replicate set from the Latin square dataset was used [14]. In this dataset fourteen specific RNAs were exogenously added to the hybridization mixture in two fold increasing concentrations. The T-test performed on this data identified all fourteen genes as well as 161 presumed false positives with a significant p-value (below 0.01). Therefore, the percentage of true positives is only 8% of the genes found significant in the result. This reflects the multiple-testing problem when using the t-test in this way. If twelve thousand tests are performed simultaneously on 12,000 genes with a type I error of 0.01 (the test is falsely considered significant one time every 100 tests), we can expect 120 (= 12,000*0.01) probe sets to be below the p-value 0.01 simply by chance. The Hochberg/Simes [15] method addresses this issue. They both found 16 genes to have a significant fold change with 11 of the 14 true positives (approx 69% true positives in the result). Another correction technique for multi-testing is the Bonferroni [15,16] method which found seven genes to be significant including six out of the fourteen true positives (approx. 86% true positives in the result). The SAM [17] method found 21 significant genes including 12 out of the 14 true positive (57% true positives in the result) for a delta of 1.54, and a Pi0Hat of 0.96. Although they were able to identify 85% of the exogenously added transcripts, their false positive rate was underestimated. SAM estimated a median false positive rate of 4.58%, but found 9 out of 21 probe-sets to be significant while they were not exogenously added (false positive rate of 43%). The Er model described above with a cut-off of 0.9 identified 12 genes with 8 of them being true positives (66% true positives in the result). These data suggest that the Er model is well within the separation levels of those standards techniques. However the results and performance of the different techniques might be dataset dependant. The replicate set from the Latin square dataset has little inherent noise. Even the chips at different control concentrations can be considered as technical replicates as only 14 out of 12,000 genes were supplemented. Using the noise model to remove noise from a noisier dataset might prove even more useful. It would be interesting to compare those methods on multiple datasets, but at the time of this study, this is the only dataset with an absolute knowledge of true and false positives. It is worth noting that the supplemented RNAs were added at a fold variance in concentration but that the actual intensity found averaged only 1.53 fold. All those methods greatly decrease the number of false positives compared to the t-test alone but some true positives were also missed. This is partly due to the fact that the control RNAs were added at concentrations testing the limits of detection.

### Cancer-specific biomarkers

The Er model was used to compare each cancer biopsy to its corresponding normal tissue. In the absence of error modeling, the directional change algorithm identified 1,910 probe-sets that had an Er score above 0.9 in ovarian cancer, 1,355 in breast cancer, 1,730 in oral cancer and 322 in prostate cancer. Incorporation of error modeling dramatically reduced the number of probe-sets with Er scores above 0.9 to 272 for ovarian, 177 for breast, 129 for oral cancer and 2 for prostate cancer [see Additional file 3]. For lung cancer biopsies, the distinct sub-classes were compared against normal tissues and 15 probe-sets with an Er value above 0.9 in all comparisons were uncovered.

The advantage of determining Er scores for differentially expressed cancer transcripts is that it provides a statistical metric that can be used to underscore markers that are unique to a particular cancer. Although the Er is not a statistical test and an Er score can vary in its significance depending on the number of samples studied, we selected genes with a high Er index in one cancer type (Er> = 0.9) and low in the others (Er<0.6). As Affymetrix HG-U95A and Hu133A contain different probe-set numbers for the same gene, the SOURCE software [18] from Stanford University was used to match the probe set to their cluster ID using the UniGene Build 167. Cluster IDs were then matched between chip types using Microsoft Access. No universal marker encompassing all the cancer vs. their normal tissue was found. This result is consistent with the result from Ramaswamy et al. [7] using 14 common tumor types including breast, prostate, ovarian and lung cancer. Nonetheless, caveolin-1 (CAV1) was found down regulated in 90% of breast, ovarian, and lung tumors, and in 80% of the prostate cancers. This gene is also down-regulated in large diffuse B-cell lymphoma [19], is associated with a region of the chromosome 7 q31 frequently deleted in tumors [20], and has been shown to have a tumor suppressing activity when restored [21,22].

The number of genes found to be reliable markers varied greatly between cancer types [see Additional File 1]. Prostate and lung cancer had the smallest number of such markers and were the 2 datasets with the most samples. The only prostate marker identified, SIM2, is a transcription factor involved in regulation of transcription during development [23]. This gene has also been found differentially expressed in colon and pancreatic cancer [24], and an antisense inhibition of *SIM2-s *expression in a colon cancer cell line restored growth inhibition and apoptotic cell death [24].

Two genes, AGER and MARCO, were found to be under expressed in all the lung cancer types compared to other cancer types. The advanced glycosylation end product-specific receptor (AGER or RAGE) has been previously reported down-regulated in non small cell lung carcinoma [25]. AGER is a receptor for amphoterin which mediates cell differentiation [26], and is highly expressed in lungs. Down-regulation of AGER may be a critical step in lung tumor formation as it is down regulated in all the different subtypes of lung cancer studied here. On the other hand, AGER seems to be up-regulated in pancreatic cancer and its level correlates to the metastatic potential of the cancer cell line [27]. The second gene specific to lung cancer is MARCO which is expressed by alveolar macrophages in the lung and is involved in inflammation and pathogen clearance [28,29]. A decrease of MARCO RNA in the sample may be due to a decrease in the number of macrophages inside the tumor compared to the normal tissue.

Thirty nine probe sets were found to have an Er score above 0.9 in ovarian cancer and lower than 0.6 for the other cancers. Two of these genes, Janus kinase 1 (JAK1) and a zinc finger homeobox (ZFHX1B), which are involved in the TGF β signaling pathway regulating cell growth, were down regulated. PAX8, a gene important in development which had been identified in an earlier study [30], was found to have consistently increased expression. Three genes involved in cell growth or maintenance, MLLT2, PRSS11, FOXO3A, were down regulated.

Breast cancer profiles have several interesting features. First, 16 ribosomal protein genes have decreased expression: L34 is involved in translational control [31], S27 in signal transduction, and RPS4X in development and cell cycle control. As the genes coding for those ribosomal proteins are located on different chromosomes the down regulation of these ribosomal proteins could be due to methylation of the ribosomal DNA [32,33]. All of the markers for breast cancer are down regulated except for inosine monophosphate dehydrogenase 1 (IMPDH1), increased by two fold, which is involved in the biosynthesis of purine nucleotides. Breast cancer has distinct sub-groups which some are hormone dependant for growth, others being very aggressive with an Her-2 amplification. The cancer samples in this study [8] are likely to be a mix of these subtypes. This might explain why the well known markers for a particular sub group do not appear in those results. However, the particular sub-classification of those 16 breast cancer samples is not known [8].

In oral cancer [see Additional File 1], many genes involved in differentiation of epithelial cells are found to be specific markers for this cancer. Keratin 4 and 13 (KRT4 and 13), and the small proline-rich protein 1B (SPPR1B) involved in epidermal differentiation, are all down regulated, as well as cellular adhesion genes desmoglein 1 and 3 (DSG1 and 3). The matrix metalloproteinase 13 (MMP13) gene encoding collagenase was specifically up regulated in this cancer. In the original study the up regulation of MMP1 and down regulation of KRT4 was confirmed by RT-PCR [34].

## Conclusions

The method described here provides improved non-parametric approaches to microarray data analysis. After applying the noise boundary model, markers were selected according to their consistency for up-regulation or down-regulation using a voting scheme comparing normal versus cancer biopsies. Tissue-specific expression differences were eliminated by comparing the cancer samples to the normal biopsies from the same tissue. The genes with the greatest differential expression between cancer and normal biopsies were then compared between cancer types. This differs from previous studies [7,8,35] which directly compare results among different cancers. Groups of markers with consistent differential expression among ovarian, breast, prostate and lung cancer were found. Many of these markers are related to de-differentiation of the tissue and were highly specific to their tissue of origin. Also, tumors arising from cells with the same embryogenic origin tend to have the same genes required for cancer progression. This confirms a previously described oncodevelopmental connection [36].

## Methods

All of the microarray data used in this analysis was derived from RNA isolated from biopsies and hybridized on Affymetrix GeneChips HG-U95A, HG-U95Av2 or HG-U133A. All the research groups used the same standard procedure for labeling the cRNA, hybridization and scanning the GeneChips [37]. The datasets were obtained from several different sources: Data from 24 breast cancer biopsies were from Su et al.[8], and the three corresponding normal breast tissue biopsies were provided by Garret Hampton from the Genomics Institute of the Novartis Research Foundation. For prostate cancer, the dataset was derived from 21 tumors and 8 normal biopsies [38] whereas the ovarian cancer dataset originated from 14 tumor and four normal biopsies [39]. Finally, the lung cancer dataset consisted of biopsies from 61 samples of lung adenocarcinoma, 20 lung carcinoids, six small cell lung cancer, 21 squamous lung cancers, and 17 normal lung tissues [40]. Out of the 61 adenocarcinoma samples, 19 were replicates and 52 were sub-divided into five categories according to Bhattacharjee et al.(2001) [40]: seven in cluster 1, nine in cluster 2, 15 in cluster 3, 13 in cluster 4, and eight samples of colon metastasis. The Oral cancer dataset consisted in 4 normal and 16 oral cancer biopsies [34]. The directional change assessment and the noise model algorithms were programmed using Python, and the comparison for markers was performed with Excel.

## Authors' contributions

VA and PT conceived the study. VA carried out the analysis and drafted the manuscript. MC performed the programming. JC helped with the evaluation of raw image analysis methods. MR supervised the programming and analysis. PS and JD participated in the study design and management.

## Supplementary Material

Additional File 2 Assessing the noise level and trust threshold for differential expression on Affymetrix GeneChips. This document compares the noise from MAS5, RMA and dChip and presents the sensitivity analysis for the noise model parameters using the Latin square replicate data set. Click here for file

Additional File 3 Gene markers for prostate, breast, ovarian, oral, and lung cancer. This file presents the Top Er scores for each cancer studied when compared to its normal tissue. There is a result table with gene information for each cancer on separate tabs. Click here for file

Additional File 1 Gene markers that distinguish between prostate, breast, ovarian, oral, and lung cancer. This file presents the Er scores of genes expression levels that are consistently up or down in a given cancer compared to its normal tissue (Er>0.9) but not in any of the other four cancers (Er<0.6). Click here for file

## Figures and Tables

**Table 1:** Average slopes and intercepts for the different tissue type. This table displays the average slope and intercept of the regression of the 80^th ^percentile of the bins by the inverse of the average expression per bin. The bin size was 200 and the minimum intensity cutoff was 100.

	Average Slope	Stdev	Average Intercept	Stdev
lung normal	96	29	1.42	0.15
Breast Normal	139	33	1.24	0.06
Ovarian Normal	154	45	1.48	0.12
Prostate Normal	61	26	1.61	0.26
Oral Normal	89	12	1.55	0.22
