# Supplementary material for: Noise filtering and nonparametric analysis of microarray data underscores discriminating markers of oral, prostate, lung, ovarian and breast cancer
Source: BMC Bioinformatics. 2004 Nov 29;5:185. doi: 10.1186/1471-2105-5-185 (PMC538261; doi:10.1186/1471-2105-5-185)
Supplement: Additional File 2 — Assessing the noise level and trust threshold for differential expression on Affymetrix GeneChips. This document compares the noise from MAS5, RMA and dChip and presents the sensitivity analysis for the noise model parameters using the Latin square replicate data set. [file 1471-2105-5-185-S2.doc]

Supplemental DATA

Assessing the noise level and trust threshold for differential expression on Affymetrix GeneChips.

## Sensitivity Analysis of the Parameters (cut off and percentile) and the Probe Set Intensity Extraction Methods

To be able to perform a sensitivity analysis on the parameters, a standard dataset was chosen. The Latin square replicate data set [1] was used for this purpose as it contains genes replicated for their expression levels and spiked in genes with different concentrations on different chips. Since the concentration of these genes was the only difference between the arrays, the true and false positive rates could be accurately determined. The 16 samples of the replicate set in the Latin square dataset were considered replicates for modeling the noise as the number of probe-sets spiked in represents only a small portion, less than 0.0012%, of the total number of probe-sets. For this replicate set, a sensitivity analysis was performed around two parameters: percentile taken for each bin for regression and minimum intensity cutoff. This sensitivity analysis was also performed for the different methods of probe set intensity extraction: dChip PM only [2, 3], MAS 5 [4] and RMA [5, 6]. The sensitivity analysis consists in observing the variation of the slope and intercept obtained from the linear regression on the boundary defined by the percentile and cut-off. The average slopes and intercepts were then graphed as a function of those two parameters.

For dChip PM only and RMA, the parameter that influences most the average slope is the minimum intensity cutoff (Figure 1 and 2). Only the highest percentiles, above 94, have an effect on the slope and decrease it drastically due to the introduction of noisy data. On the other hand, the slopes increase steadily with the minimum intensity cutoff for RMA and with a delay for dChip PM only. This can be due to non linear increase of the absolute fold change with the decrease in bin intensity. The increase has concave shape, the more the low end data is cut, the higher the slope of the regression. Also as an artifact of the transformation of the x-axis, inverse of the average bin intensity, there is also a non-equidistant repartition of the bin percentile fold change. This result in bin with a low average intensity having more weight on the regression than the bins with higher intensities: i.e. more bins are regrouped in the 0 to 0.02 range than 0.02 and 0.04 range. As the average intensity gets higher, the differences of the fold change percentiles decrease and the inverse of the average intensity decreases further. Eliminating the low intensity bins has a greater effect on the slope. Also, slopes with a cut-off higher than 500 are probably not reliable as the regressions were performed on less than 20% of the original data.

**Figure 1**  Three dimensional graph of the effect of the minimum intensity cutoff and percentile on the slope of the regressed percentile to the average intensity of the bins, with data obtained with the RMA [6].

**Figure 2** Three dimensional graph of the effect of the minimum intensity cutoff and percentile on the slope of the regressed percentile to the average intensity of the bins, with data obtained with the dChip PM only.

In Figure 3, MAS 5 average derived slopes are both influenced by the minimum intensity cutoff and the percentile. The slope increases in a step like manner increasing with a larger percentile and a larger minimum intensity cutoff. The magnitude of the slope is also very different between MAS 5, dChip PM only and RMA. For the 80^th^ percentile and a minimum intensity cutoff of 100, the average slope is 157.6 for MAS 5, 7.7 for dChip PM only and 2.15 for RMA. Since the regression is performed on the inverse of the average intensity, the slope gives an indication of the noise in the low intensity range. Because the Latin square GeneChips can be considered as replicates, it can be inferred that RMA and dChip PM only are better controlling the noise in the low intensity range compared to MAS 5. One explanation could be the fact that dChip PM only and RMA attenuates the signal compared to MAS5, reducing both noise and true fold change (unpublished data). In all three cases there are conditions where the slope is stable for minor variations of the cutoff or percentile.

**Figure 3** Three dimensional graph of the effect of the minimum intensity cutoff and percentile on the slope of the regressed percentile to the average intensity of the bins, with data obtained using MAS5 (Affymetrix).

For all three methods of probe-set intensity extraction, the average intercept is insensitive to the percentile and the minimum value cutoff except for the extreme values of those parameters: i.e. 98 percentile and no minimum value cutoff (see Figures 4, 5, 6). For the 80^th^ percentile and a minimum intensity cutoff of 100, the average intercept is also very similar between the methods: 1.08 for MAS 5, 1.13 for dChip PM only and 1.12 for RMA.

**Figure 4** Three dimensional graph of the effect of the minimum intensity cutoff and percentile on the intercept of the regressed percentile to the average intensity of the bins, with data obtained using RMA [6].

**Figure 5** Three dimensional graph of the effect of the minimum intensity cutoff and percentile on the intercept of the regressed percentile to the average intensity of the bins, with data obtained using dChip PM only.

**Figure 6** Three dimensional graph of the effect of the minimum intensity cutoff and percentile on the intercept of the regressed percentile to the average intensity of the bins, with data obtained using MAS5 (Affymetrix).

To evaluate the performance of the noise boundary model to identify the two fold change spiked-in genes in the results and eliminate false positives from the results, the sum of the rank of 12 out of the 14 spiked probes was evaluated. Two out of the 14 spiked genes were omitted because either their concentration was too low to be detectable (1597_at was spiked at the concentration of 0 to 0.25 pm) or the amount had saturated (1708_at was spiked at 256 and 512 pm). The noise boundary model was applied to any combination of the chips spiked at one concentration to all the other chips spiked at the other concentration. For every gene, up-regulation or down-regulation was then recorded as the fold change was compared to the noise boundary. The maximum number of fold change directions was then divided by the number of comparisons. Those probe-sets were ranked in descending order according to their Er score (Highest Er score is assigned the number 12626: i.e. the number of genes in the chip). The scores obtained were summed for the 12 spiked probe-sets and the results were normalized. The perfect score is 1. The result with the probe signal estimated with MAS 5 is presented in Figure 7. A plateau can be observed at 0.99 for most of the range for percentile and cutoff values. However there is a sharp decrease for a low cutoff, i.e. zero. The percentile also had little effect until a percentile higher than 94% was reached. The high percentile and low cutoff introduce more noise in the data setting the noise boundary model too high, therefore reducing the Er score of the spiked-in genes. The graph (Figure 8) for the signal estimation with RMA is similar to the MAS 5 in that most of the area covered by the simulation for the percentile and cutoff is a plateau at 0.99 for the sum of rank of the spiked in genes. In the same manner, the sum of ranks decreases for higher percentiles (above 96%). However, in this case, the rank is insensitive to low intensity cutoffs but decreases with higher cutoffs, with a first dip with gene intensities lower than 1600, and a second for intensities of 2100. The higher cutoffs are actually cutting most of the data to construct the model. For perspective, the chips are scaled to an average intensity of 300.

The dChip PM only algorithm did not seem to perform as well as MAS 5 and RMA, as its plateau was smaller and more sensitive to the parameters (Figure 9). The plateau average is also 0.99, but it is limited to percentiles lower than 90% and a minimum intensity cutoff lower than 1100. Overall, MAS 5 seemed to be more robust, as the noise boundary model performance gradually decreased with the percentile.

**Figure 7** Three dimensional graph of the effect of the minimum intensity cutoff and percentile on the score of the spiked in genes of the replicate set of the Latin square dataset, with data obtained using MAS5 (Affymetrix).

**Figure 8** Three dimensional graph of the effect of the minimum intensity cutoff and percentile on the score of the spiked in genes of the replicate set of the Latin square dataset, with data obtained using RMA [6].

**Figure 9** Three dimensional graph of the effect of the minimum intensity cutoff and percentile on the score of the spiked in genes of the replicate set of the Latin square dataset, with data obtained using dChip PM only.

A compromise has to be made between setting a noise boundary model to low, finding all the spiked genes with a lot of false positives and setting the noise model too high. At this point the model is so conservative that only a few spiked in genes are found. Figure 10 displays the false positive rate when an Er cutoff is set to 0.9, equivalent to a gene being consistently over or under expressed in 90% of the comparisons. The false positive rate is very high for a percentile of 0.98. This artifact is due to the presence of only one gene which is a false positive in the result set. The false positive rate decreases sharply with the percentiles and then increases again as the boundary model becomes less conservative.

**Figure 10** Three dimensional graph of the effect of the minimum intensity cutoff and percentile on the false positive rate of the genes with an Er index above 0.9 in the replicate set of the Latin square dataset, data obtained using MAS5 (Affymetrix).

## Sensitivity Analysis Discussion

Noise modeling can be performed for all of the methods. MAS 5 was shown to be noisier for low intensities but the correction using the noise model seemed to perform very well as its performance decreased gracefully for finding spiked genes. MAS 5 will be used in further studies because of its wide use for signal intensity estimation and its robustness for the two parameters tested. A cutoff for the minimum intensity of 100 and the 80^th^ percentile was selected for the model parameters as they are in regions where the slope, intercept and rank are not very sensitive to change, and where the false positive rate is reasonable.

## Evaluation of the Noise Model on Real Data

Figure 11 displays the 80^th^ percentile error boundaries for five different normal tissues as a function of the inverse average bin intensity. Bins with an average intensity lower than 100 (above 0.01 in the Figure) were not displayed. They are below the minimum intensity cutoff and hinder the linearity relation of the percentile to bin intensity. A leveling off of the fold changes at high was also noticed; this leveling is due to saturation on the chip. To decrease the effect of the saturation on the regression, the top 8% of the genes were eliminated i.e. top 5 bins with lowest inverse average intensity. The slope and intercept were then calculated for each cancer dataset as they give an indication of the noise level at low and high expression values respectively. For each comparison of normal samples in a tissue, the slope and intercept were averaged (Table 1). There seems to be a negative correlation between the slopes and intercepts. The higher the intercept the lower the slope. If a dataset contains an inherent high background, the signal to noise ratio is decreased. The intercept will increase as the 80^th^ percentile is going to be higher. The slope on the other hand is not going to increase, and might even decrease as the low intensity background noise remains constant. Before using this noise boundary model in to find cancer markers, the stability of the slope and intercept for the different datasets must be evaluated. One of the differences with the Latin square replicate data set is that these public data consist of biological replicates of normal tissue instead of technical replicates. The cancer biopsies were not used in designing the noise model as they might be more variable than normal tissue. The first two simulations were then performed for all normal tissue samples to confirm that the minimum intensity cutoff and percentile selected were also in regions where their slope was also insensitive to small changes (Figures 12, 13, 14, 15, 16). Figures for the simulation of the effect of the cut-off value and percentile on the intercepts are presented in Appendix B. For all the normal tissue, the results from the simulation are very similar.

**Figure 11** This Figure represents the 80^th^ percentile for each of the five tissues plotted against the inverse of the average bin intensity. The different normal tissues are represented in color, ▲ for breast, * for Ovarian, x for Prostate, ■ for Oral and ♦ for lung.

**Table 1** Average Slopes and Intercepts for the Different Tissue Types

This table displays the average slope and intercept of the regression of the 80^th^ percentile of the bins by the inverse of the average expression per bin. The bin size was 200 and the minimum intensity cutoff was 100.

|  | Average Slope | Stdev |  | Average Intercept | Stdev |
| --- | --- | --- | --- | --- | --- |
|  |  |  |  |  |  |
|  |  |  |  |  |  |
| Lung normal | 96 | 29 |  | 1.42 | 0.15 |
|  |  |  |  |  |  |
| Breast Normal | 139 | 33 |  | 1.24 | 0.06 |
|  |  |  |  |  |  |
| Ovarian Normal | 154 | 45 |  | 1.48 | 0.12 |
|  |  |  |  |  |  |
| Prostate Normal | 61 | 26 |  | 1.61 | 0.26 |
|  |  |  |  |  |  |
| Oral Normal | 89 | 12 |  | 1.55 | 0.22 |

**Figure 12** Three-dimensional graph of the effect of the minimum intensity cutoff and percentile selected on the slope of the regressed percentile to the average intensity of the bins for the prostate normal biopsies.

**Figure 13** Three-dimensional graph of the effect of the minimum intensity cutoff and percentile selected on the slope of the regressed percentile to the average intensity of the bins for the lung normal biopsies.

**Figure 14** Three-dimensional graph of the effect of the minimum intensity cutoff and percentile selected on the slope of the regressed percentile to the average intensity of the bins for the ovarian normal biopsies.

**Figure 15** Three-dimensional graph of the effect of the minimum intensity cutoff and percentile selected on the slope of the regressed percentile to the average intensity of the bins for the breast normal biopsies.

**Figure 16** Three-dimensional graph of the effect of the minimum intensity cutoff and percentile selected on the slope of the regressed percentile to the average intensity of the bins for the oral normal biopsies.

## Conclusion

There is a characteristic increase in the range of fold change values that occur at lower expression levels on Affymetrix GeneChips replicate arrays. This occurs with all techniques for estimating gene expression levels from GeneChips measurements. This increase in fold change was found to be consistent and could be characterized through regression analysis in the three different, commonly used, probe set intensities extraction methods (MAS5, dChip PM only and RMA). This noise was also shown to be consistent not only on replicate arrays but also on normal tissue replicate data. The boundary of this noise can be modeled for all data extraction methods and is found to fit well with an inverse linear function. From this observation, a noise boundary model was derived.

**References:**

1. E Hubbell, WM Liu, R Mei: **Robust estimators for expression analysis**. *Bioinformatics* 2002, **18**:1585-92.

2. C Li, WH Wong: **Model-based analysis of oligonucleotide arrays: expression index computation and outlier detection.** *Proc Natl Acad Sci USA* 2001, **98**:31 - 36.

3. C Li, WH Wong: **Model-based analysis of oligonucleotide arrays: model validation, design issues and standard error application.** *Genome Biol* 2001, **2**:research0032.1 - 0032.11.

4. Affymetrix: **Affymetrix Microarray Suite 5.0 User's Guide**. *Affymetrix, Santa Clara* 2001.

5. RA Irizarry, B Hobbs, F Collin, YD Beazer-Barclay, KJ Antonellis, U Scherf, TP Speed: **Exploration, normalization, and summaries of high density oligonucleotide array probe level data.** *Biostatistics* 2003, **4**:249-264.

6. R Ihaka, R Gentleman: **R: A Language for Data Analysis and Graphics**. *Journal of Computational and Graphical Statistics* 1996, **5**:299-314.
